# Supplementary material for: Nucleation Points: The Forgotten Parameter in the Synthesis of Hydrogel-Coated Gold Nanoparticles
Source: Polymers (Basel). 2021 Jan 26;13(3):373. doi: 10.3390/polym13030373 (PMC7865208; doi:10.3390/polym13030373)
Supplement: Supplementary file 1 [file polymers-13-00373-s001.pdf]

†Electronic Supplementary Information (ESI)

Nucleation points: The forgotten parameter in the synthesis of hydrogel-coated gold nanoparticles

Adolfo Sepúlveda,[a,c] Audrey Picard-Lafond,[b,c] André Marette[d] and Denis Boudreau[b,c]*

[a]Département de biochimie, de microbiologie et de bio-informatique, Université Laval, Québec, Québec G1V 0A6, Canada.

[b]Département de chimie, Université Laval, Québec, Québec G1V 0A6, Canada.

[c]Centre d'optique, photonique et laser (COPL), Université Laval, Québec, Québec G1V 0A6, Canada.

[d]Département de médecine, Faculté de médecine, Institut universitaire de cardiologie et de pneumologie de Québec (IUCPQ) et Institut sur la nutrition et les aliments fonctionnels (INAF), Université Laval, Québec G1V 0A6, Canada.

*E-mail: Denis.Boudreau@chm.ulaval.ca

Functionalization of gold nanoparticles:

Table S1 : Volume of 1-mM SDS and 1.4-mM B-en-A solutions used depending on diameter of starting gold cores.

Gold core diameter (nm)	SDS (mL)	B-en-A (mL)
15	3.00	1.64
35	4.72	2.58
50	6.00	3.26

TEM and size distribution of Au@pNIPAM nanoparticles:

S1: 50-nm AuNPs@pNIPAM

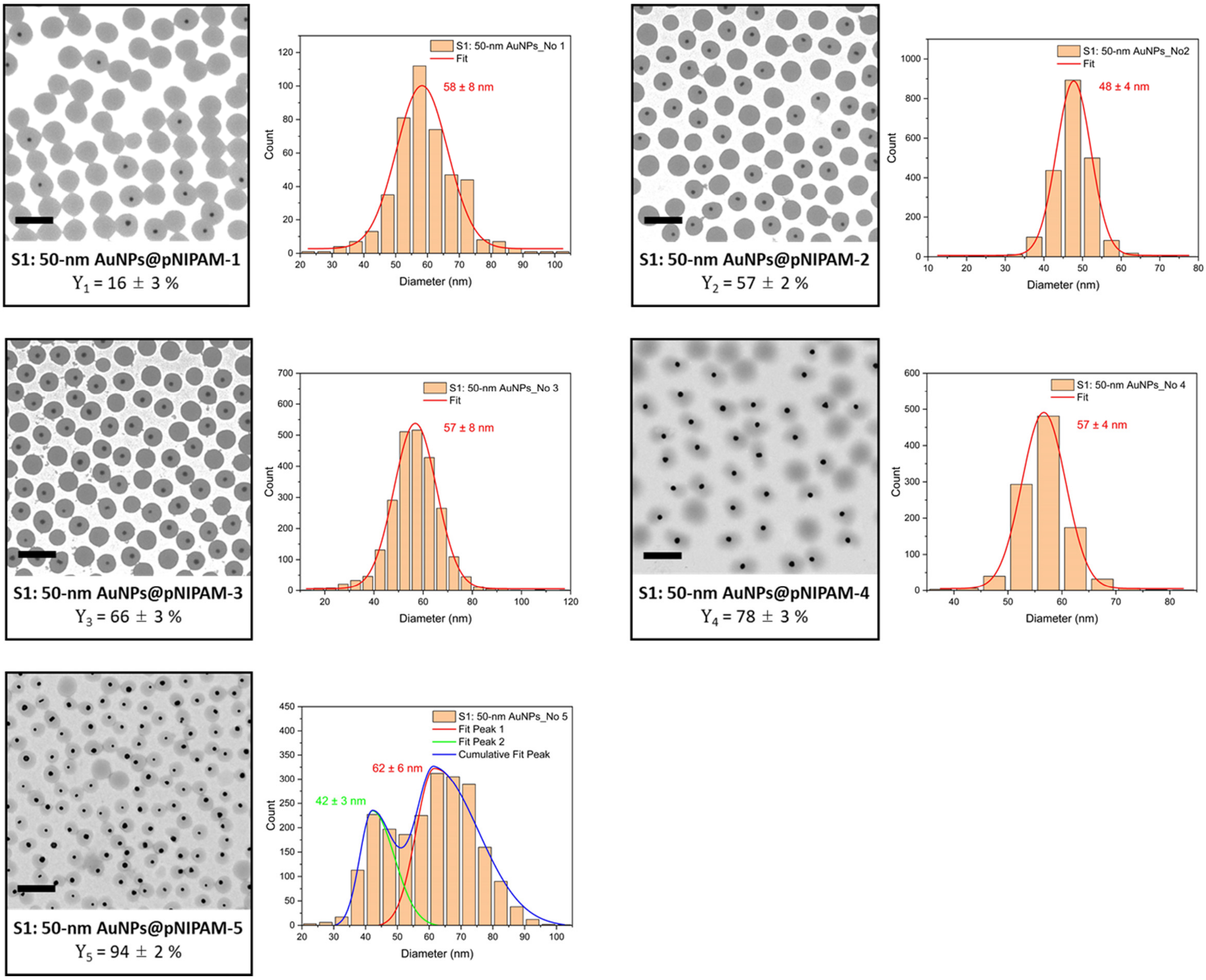

Figure S1 : Transmission electron micrographs of S1 polymerization series and their corresponding size distribution histograms of gold cores. Scale bars are 500 nm.

S2: 35-nm AuNPs@pNIPAM

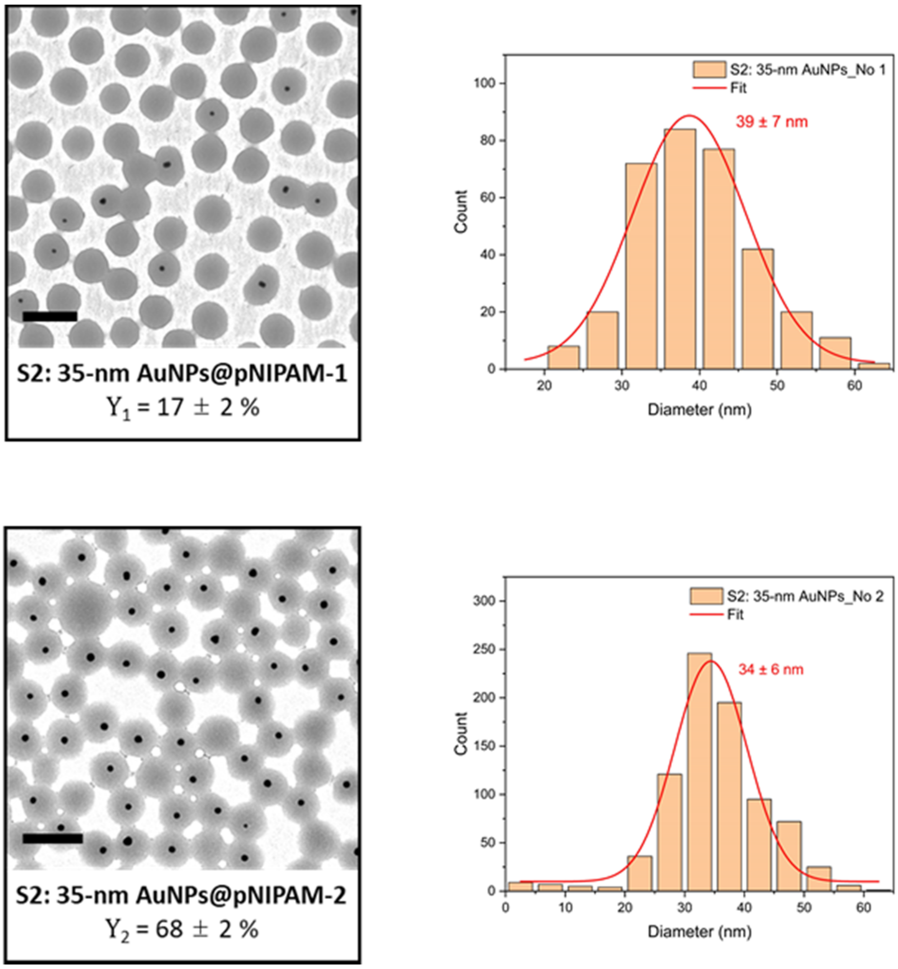

Figure S2 : Transmission electron micrographs of the S2 polymerization series and their corresponding size distribution histogram of the gold cores. Scale bars are 500 nm.

S3: 15-nm AuNPs@pNIPAM

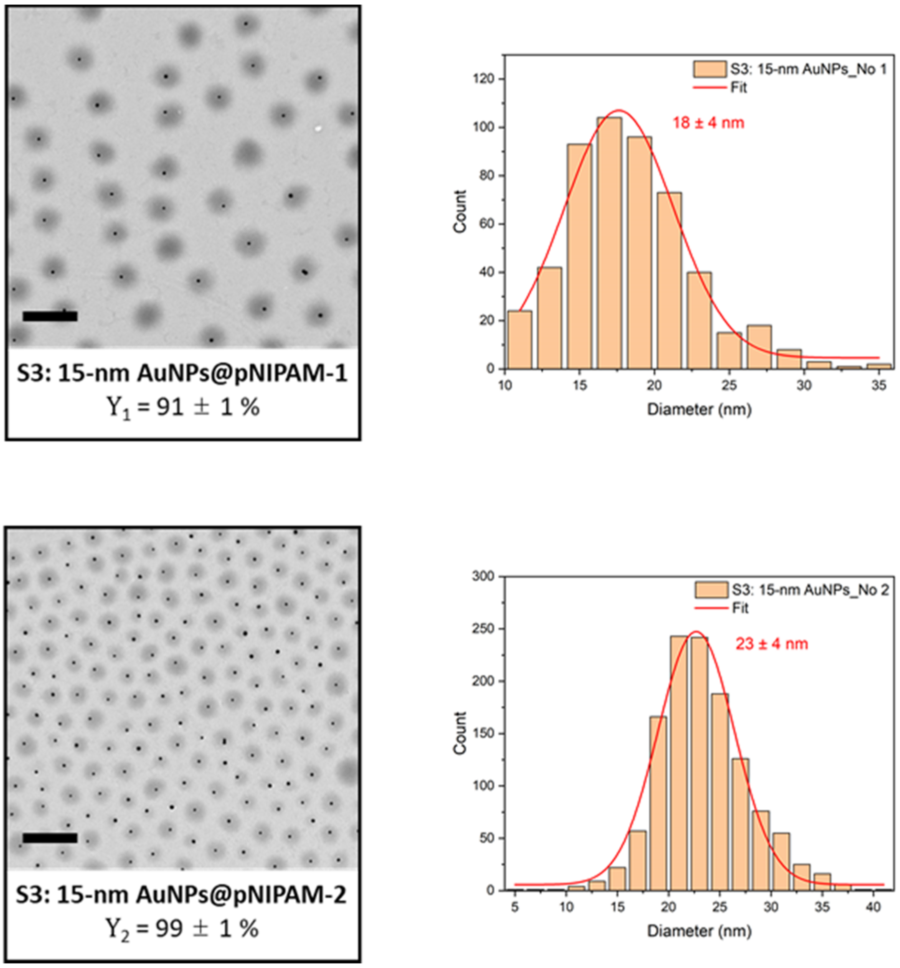

Figure S3 : Transmission electron micrographs of the S3 polymerization series and their corresponding size distribution histogram of the gold cores. Scale bars are 500 nm.

Bimodal samples - S4: 50/65-nm AuNPs@pNIPAM and S5: 25/65-nm AuNPs@pNIPAM

Table S2 : Number of functionalized gold nanoparticles utilized in both bimodal samples S4 and S5.

Sample	Number of 25-nm AuNPs (x10^{12})	Number of 50-nm AuNPs (x10^{12})	Number of 65-nm AuNPs (x10^{12})
S4: 50/65-nm AuNPs@pNIPAM	-	4.4 ± 0.1	1.7 ± 0.1
S5: 25/65-nm AuNPs@pNIPAM	8.0 ± 0.3	-	6.6 ± 0.4

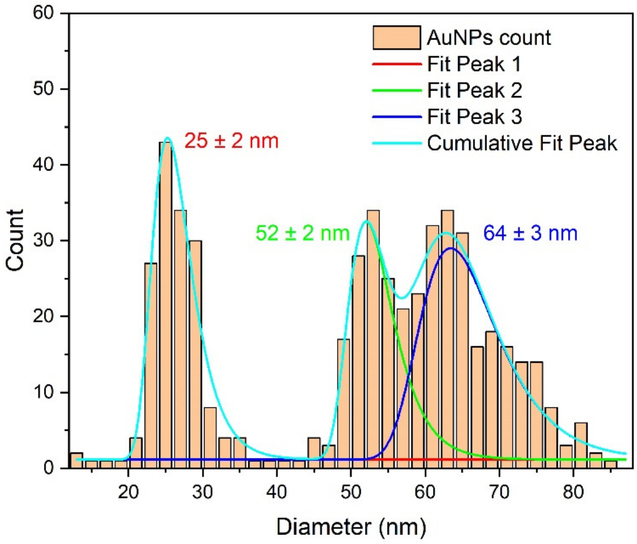

Figure S4 : Size distribution histogram of the gold cores employed in both bimodal samples S4 and S5.

Preparation of pNIPAM microgels without metallic cores

Spherical microgels of pNIPAM were synthesized following the previously explained precipitation polymerization protocol but omitting the step of adding the functionalized gold nanoparticles. Hence, 57 mg of NIPAM and 12 mg of BIS were dissolved in 25 mL of nanopure water at room temperature and under continuous stirring (600 rpm). After complete dissolution, the mixture was heated to 70 °C and purged with nitrogen to remove oxygen. The polymerization was initiated after 20 min by the rapid addition of 1 mL of an aqueous KPS solution (1.85 mM) and allowed to proceed for 2 h. Afterward, the solution was cooled down to room temperature and purified by three consecutive centrifugations at 6000 rcf until obtaining a clear supernatant. The resulting pNIPAM microgels were redispersed in 5 mL of nanopure water and stored at 4 °C.

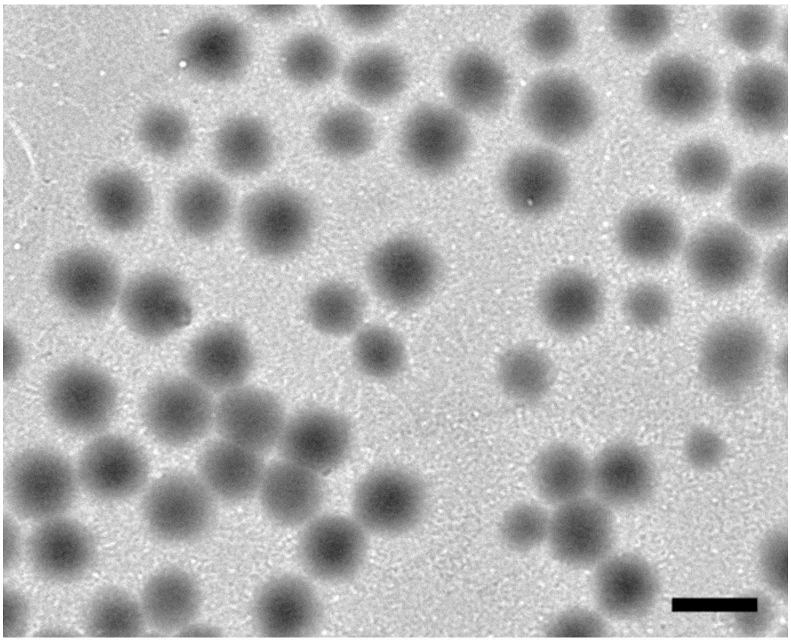

Figure S5 : Transmission electron micrograph of pNIPAM microgels without metallic cores. Scale bar is 500 nm.

Overall particle surface and encapsulation yield

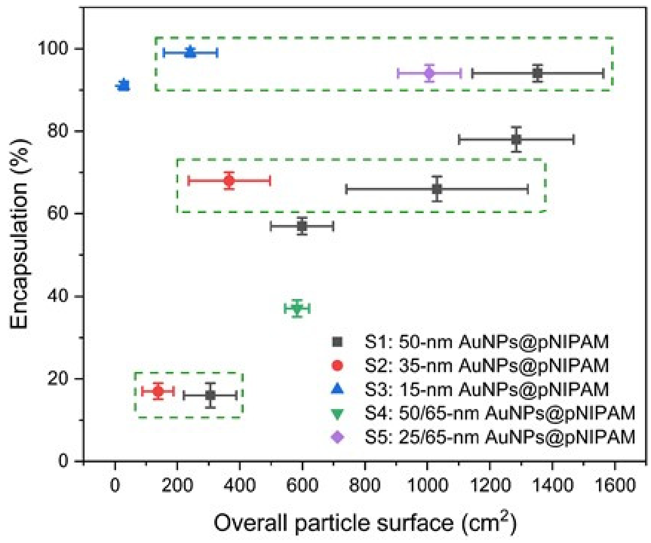

Figure S6 : Resulting yield as a function of the overall particle surface calculated for each sample.

Polydispersity index (PDI)

Table S3 : Summary of the particle hydrodynamic diameters and their polydispersity indices measured by DLS. ‡

Samples	T = 15 °C		T = 50 °C	
	D_H (nm)	PDI	D_H (nm)	PDI
S1: 50-nm AuNPs@pNIPAM-1	501 ± 6	0.015 ± 0.001	339 ± 5	0.049 ± 0.037
S1: 50-nm AuNPs@pNIPAM-2	370 ± 3	0.007 ± 0.007	255 ± 1	0.009 ± 0.008
S1: 50-nm AuNPs@pNIPAM-3	391 ± 7	0.046 ± 0.055	264 ± 8	0.020 ± 0.009
S1: 50-nm AuNPs@pNIPAM-4	345 ± 1	0.005 ± 0.005	230 ± 3	0.024 ± 0.006
S1: 50-nm AuNPs@pNIPAM-5	231 ± 4	0.012 ± 0.011	146 ± 3	0.074 ± 0.023
S2: 35-nm AuNPs@pNIPAM-1	378 ± 2	0.011 ± 0.005	254 ± 6	0.024 ± 0.013
S2: 35-nm AuNPs@pNIPAM-2	372 ± 3	0.029 ± 0.018	253 ± 4	0.017 ± 0.004
S3: 15-nm AuNPs@pNIPAM-1	305 ± 5	0.008 ± 0.006	204 ± 3	0.014 ± 0.011
S3: 15-nm AuNPs@pNIPAM-2	188 ± 4	0.039 ± 0.019	120 ± 1	0.055 ± 0.009
pNIPAM microgels	331 ± 6	0.027 ± 0.024	243 ± 5	0.011 ± 0.004

‡The uncertainties correspond to the standard deviation of the mean values measured by DLS (N=3).

Shrinking ratio

The shrinkage capacity for each sample was calculated using the following equation [1]:

$$\text{Shrinking ratio} = \left(\frac{\mathrm{R_{shrunken}}}{\mathrm{R_{swollen}}}\right)^3 \times 100\% \qquad (1)$$

Here, $\mathrm{R_{shrunken}}$ corresponds to the mean hydrodynamic radius of the gold-pNIPAM core-shell nanoparticle measured at the shrunken state (50 °C). $\mathrm{R_{swollen}}$ is the mean hydrodynamic radius measured at the swollen state (15 °C).

[1] Hellweg T, Kratz K, Pouget S, Eimer W. Internal dynamics in colloidal PNIPAM microgel particles immobilised in mesoscopic crystals. vol. 202. 2002.
